# Research on model design and operation mechanism of enterprise blockchain digital system

**DOI:** 10.1038/s41598-022-24796-0

**Published:** 2022-11-24

**Authors:** Xin Su, Shengwen Wang

**Affiliations:** School of Business Administration, Shandong University of Finance and Economics, Jinan, 250014 China

**Keywords:** Engineering, Electrical and electronic engineering

## Abstract

Emerging technologies such as blockchain have accelerated the digitization of a variety of industries, improved the operational efficiency of enterprises, and promoted in-depth integration of digital technology with the real economy. Blockchain has characteristics that include distributed storage, peer-to-peer transmission, strong confidentiality, and easy traceability. This article introduces blockchain into an enterprise’s information management system with the aim of breaking the enterprise’s digital barriers by using technologies such as distributed ledgers, smart contracts, and asymmetric encryption, thus improving the security and applicability of the enterprise data assets. This article explores the characteristics and security of three types of blockchain in depth, designs the model framework of the blockchain digital system (BDS) based on industry needs, and analyzes the functions and the operating mechanisms of each level of the system in detail. Finally, based on the characteristics of public blockchain, consortium blockchain, and private blockchain, three typical application scenarios in which the BDS can be used are selected, and the article discusses how E-retail supply chains, virtual power plants, and carbon trading platforms can realize digital management using the BDS, thus providing a practical basis for construction and application of the BDS.

## Introduction

**Figure 1:**
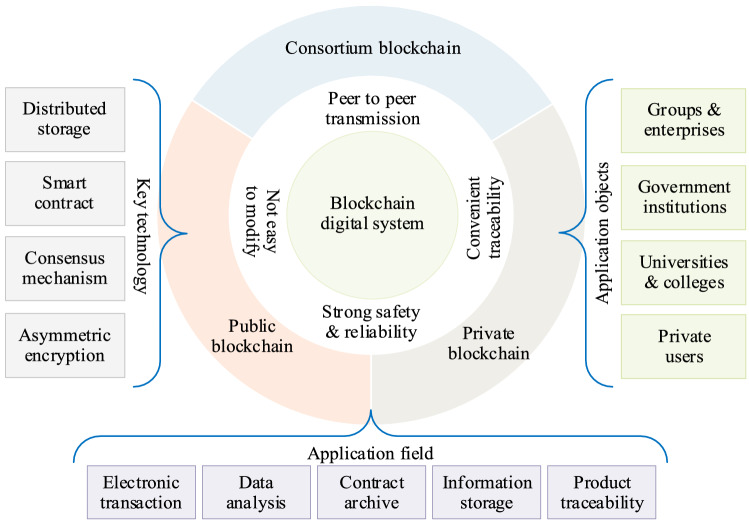
Attribute characteristics of blockchain.

The continuous innovation provided by blockchain, the Internet of things (IoT), artificial intelligence (AI), and other digital technologies has promoted the development of Industry 4.0, and if enterprises want to improve their competitiveness continuously, they must accept and embrace the megatrend of digital transformation^1^. At the same time, the sudden outbreak of the Covid-19 pandemic has also accelerated the application of digital technology, with many industries and individual companies gradually realizing that application of digital technology such as blockchain is related not only to business operating efficiency, but also to the actual survival of these enterprises^2^. To truly realize the landing application of blockchain technology, enterprises should not stop at the marketing and management level, but should also implement the required learning and reforms related to digital technology systematically. The blockchain concept first appeared in Satoshi Nakamoto’s paper “Bitcoin: A Peer-to-Peer Electronic Cash System” in 2008, and the technology was first applied to Bitcoin and virtual currency trading systems^3^. Blockchain integrates basic technical elements such as distributed storage, peer-to-peer networks, consensus mechanisms, and smart contracts (as shown in Fig. 1) to form a new method of data recording, storage, and transmission, and solve technical problems such as decentralization, openness, transparency, and high-level autonomy^4^.

Based on an analysis of the blockchain characteristics, this article proposes that this technology can drive digital transformation of enterprises from the following perspectives. (1) Building a new digital transaction model. A blockchain can be regarded as an electronic database where each node in the blockchain network stores the complete data information in the network. This structure breaks away from the traditional centralized structure and forms a distributed storage system. The blockchain makes it difficult to tamper with the data stored in the system, ensures that the data are authentic and reliable, and then helps partners who do not know each other establish trust relationships based on these trusted data. This new trust model built using blockchain can help companies to adapt more readily to the anonymous cooperation model in the digital world, cooperate more with unfamiliar partners, and provide better services to unknown customers. (2) Strengthening the data management application capability. Each node in the blockchain stores all the data on the system, which helps companies to solve the “data silos” problem, makes each working process open and transparent, and improves collaboration efficiency. The asymmetric encryption technology that is integrated within the blockchain realizes safe sharing of information without revealing any private data. Therefore, blockchain helps enterprises to manage various process data more securely during the digital transformation process. (3) Integration of smart contracts to improve workflow efficiency. Businesses can write predetermined conditions and terms into blockchain smart contracts in the form of code. When these conditions or terms are met, the smart contract automatically begins to execute, thereby increasing the process efficiency. Based on the fact that blockchain data are difficult to modify illegally, the execution of smart contracts is also irreversible and these contracts are difficult to adjust, thus ensuring the safety and reliability of process execution.

By considering blockchain from the perspectives of classification and security as a starting point, this article performs a detailed analysis of the pain points of enterprises in terms of data value mining and use, constructs the model framework of the blockchain digital system, and demonstrates how in-depth integration of digital technology with industrial entities can be realized through blockchain. Overall, the main contributions of this study are described as follows.

- Exploration of the types and characteristics of blockchain and its security.
- Design of a platform framework for the blockchain digital system (BDS).
- Demonstration of the operating mechanism of the BDS in three typical application scenarios.

## Related research

### Literature review of blockchain

As a result of the gradual upgrading of technology, massive amounts of data are generated during operation of an enterprise^5^. However, because these data commonly come from different businesses and departments, the data formats and attributes are also different, and they have complex, multi-source, and heterogeneous characteristics. When transmitting and sharing these data, so-called information island problems will then be generated^6^. As an emerging industrial technology, blockchain has characteristics that include distributed storage, peer-to-peer (P2P) transmission, and asymmetric encryption, and these characteristics are expected to alleviate these enterprise data asset management and application problems^7^. In short, blockchain, as an open information recording system, is a distributed database that is jointly maintained by its different nodes. The blockchain is composed of data blocks that are generated by cryptography; each block is stamped with a timestamp, and each has a private key generated by a hash value. Each block also contains the private key of the previous block, with the blocks being linked from the genesis block up to the current block and thus finally forming the blockchain^8^.

With the advent of the 5G era, financial technology companies were the first to discover that the distributed ledger and asymmetric encryption technologies of blockchain have strong application value for securities industries. As blockchain continues to mature, the technology is widely used in the IoT, communications, energy, medicine, and other fields. To eliminate the poor environmental adaptability of the industrial IoT (IIoT) and the problem that equipment can easily be hijacked, Feng built a consortium blockchain-based access control framework for 5G-enabled IIoT to improve device throughput^9^, and Wang proposed a novel pairing-free certificateless scheme that uses advanced blockchain technology and smart contracts to build a novel and reliable certificateless signature scheme^10^. As a technical extension of the IoT, blockchain has also promoted the development of the Internet of Vehicles (IoV). Su developed a blockchain privacy protection system for the IoV. This system designed a set of two-way authentication and key agreement algorithms using the signature algorithm, which solved the central dependence problem of the traditional IoV system^11^. Additionally, blockchain has also been applied in the electronic communications field. Khan found that by using blockchain and 6G technologies, network decentralization and resource sharing can maximize resource utilization and improve the reliability of ultra-long-distance communications^12^. To reduce the effects of proof of work (PoW) on the communication network, Li designed a blockchain-based mobile edge computing (MEC) system to solve the PoW problem^13^. In the energy field, use of the blockchain power system has been gradually promoted in energy projects. Chen upgraded the structure of a smart grid data platform with the aid of blockchain by analyzing the characteristics of the grid data^14^. In the medical field, health monitoring equipment is convenient for doctors and patients, but the open communication channels have both security and privacy risks. For this reason, Wang designed a lightweight wireless medical sensor network authentication protocol that combines blockchain technology and physically unclonable functions^15^.

In summary, this study has found that the technical application characteristics of blockchain are closely compatible with the digital strategy for enterprises. Therefore, to promote the feasibility of scientific achievement of the BDS, this research mainly supplements the existing literature from the following three perspectives: (1) analysis of the different blockchain classifications and their security in practical applications; (2) exploration of the framework model and the operating mechanism of enterprise BDS; and (3) demonstration of the applicable industry fields and scenarios for the enterprise BDS.

### Classification of blockchain

Essentially, blockchain refers to information processing technology that packages information that must be recorded into blocks through specific algorithm programming techniques and then uploads these blocks into distributed storage nodes, ultimately forming a chain database. This technology is characterized by decentralization and easy traceability, and it is difficult to forge. Blockchain can encrypt, transmit, and store data without reliance on any third party. Blockchain can be divided into the following three categories based on its access mechanism: public blockchain, consortium blockchain, and private blockchain^16^. A public blockchain is open to the entire network and can be accessed by any user. A consortium blockchain retains part of the centralized control function, and its data processing function and scalability are strong. A private blockchain is a private and exclusive network structure (see Table 1 for the characteristics of these three blockchain types). The different technical characteristics of blockchain make it highly suitable for use in the construction of digital management systems for various enterprises.

**Table 1:** Characteristics of the three blockchain types.

Characteristics	Public blockchain	Consortium blockchain	Private blockchain
Openness	Fully public	Partly public, partly encrypted	Fully encrypted
Degree of centralization	Fully decentralized	Retains some centralized functions	Fully centralized
Scope of application	Widest	Wider	Narrowest
Participants	Anyone	Pre-designated personnel only	Managers only
Data read and write	All participants	Persons with special permissions only	Managers only
Confidentiality	Weakest	Strong	Strongest
Features	Completely decentralized, reliable, and data are open, transparent, and unforgeable	Partially decentralized, ability to set up special management authority, strong scalability, high information processing speed, and low transaction costs	Easy to control, convenient rule modification, and offers efficient management
Application scenarios	Virtual currency transactions	Digital transformation of enterprises, and information transmission, storage, and management	Accounting audit

### Security analysis of blockchain

Within the technical framework of the blockchain, all nodes in an enterprise digital management system can participate in data processing, and the network nodes jointly verify the legality of all data transactions. The BDS is not reliant on any trusted institutions or on tripartite intermediaries when the program is running, and even if some nodes are attacked illegally, these attacks do not disrupt the smooth operation of the entire system. In addition, the digital system relies on the distributed storage, P2P transmission, asymmetric encryption, and smart contract characteristics of blockchain to ensure the unforgeable nature and traceability of its data.

However, the BDS still faces a number of security issues. Previous studies have shown that a hacker’s attack process on the digital system has the characteristics of a Poisson distribution, and the length of the data block chain ultimately determines whether or not the block information can be authenticated successfully^17^. If the probability that the real node in the BDS will obtain the accounting right is *p*, the probability that the attacking node will obtain the accounting right is *q*, and *p* + *q* = 1, then the probability *P*_*n*_ that an attacker can ultimately eliminate the gap between *n* blocks can be expressed as:

1 $$Pn = \left\{ \begin{gathered} (\frac{q}{p})^{n} , \, p > q \hfill \\ \, 1, \quad\quad \, p \le q \hfill \\ \end{gathered} \right.$$

The extended length of the blockchain of the hacker attacking the enterprise digital system satisfies the characteristics of a Poisson distribution, and its expected value is:

2 $$\lambda = n\frac{q}{p}$$

Assuming that a normal data transaction links *n* blocks, the attacker must then imitate the normal authentication method during this period to launch an attack on the BDS, and the probability that the hacker can tamper with the transaction successfully by forging the transaction node *P*_*k*_ is:

3 $$P_{k} = \sum\limits_{k = 0}^{\infty } {\frac{{\lambda^{k} e^{ - \lambda } }}{k!}} \cdot \left\{ \begin{gathered} (\frac{q}{p})^{n - k} {, }k \le n \hfill \\ \, 1,\quad\quad \, k > n \hfill \\ \end{gathered} \right.$$

**Figure 2:**
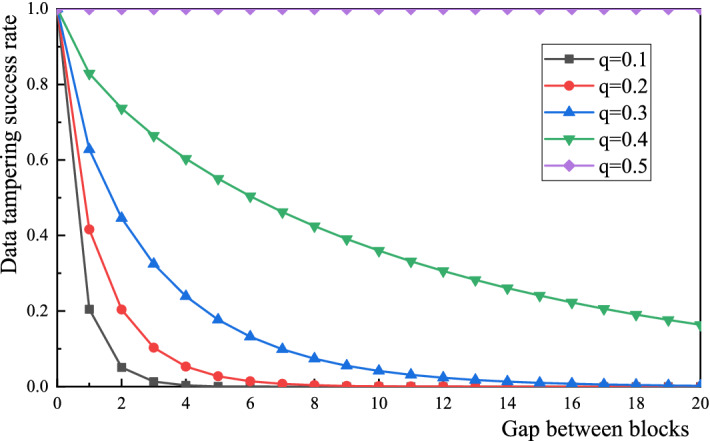
Data tampering success rate characteristics for the BDS.

The mathematical software package Maple can calculate the probability *P*_*k*_ that a counterfeit transaction node will tamper successfully with the data, and the drawing software package Origin is used to simulate the digital system tampering success rate. The experimental results are shown in Fig. 2 (Source code and data are online available at https://github.com/sw8258/BDSsourcecode.git). The data in Fig. 2 show that when the probability *q* that an attacking node will obtain the accounting right remains constant and the block gap *n* gradually increases, then the success rate of illegal tampering with the digital system will gradually decrease in tandem. When the block gap remains constant and the probability *q* that the attacking node will obtain the accounting right increases, then the probability that the system will be modified illegally also increases^18^. When the probability that the attacking node obtains the accounting right is 0.5 (i.e., *q* = 0.5), it is then possible to tamper with the system data, regardless of the value of the block gap. This demonstrates that if a hacker wants to modify the system data illegally, then they must crack more than half of the system’s block nodes simultaneously, i.e., they must launch a “51% attack” on the blockchain. However, in terms of cracking technology, it is difficult to launch a 51% attack on the blockchain without being noticed by maintenance personnel. Furthermore, it is almost impossible to tamper with the information on the blockchain at the current computing level of conventional computers because the mainstream data analysis algorithm used in the blockchain’s smart contract layer is the SHA-512 algorithm; the output space of this algorithm is (2)^512^ and it has a very strong anti-hijacking ability. Even if the current computing power of the Sierra supercomputer was used, it would take (2)^32^ years to crack the SHA-512 algorithm. Therefore, when the scale of the enterprise data is large enough, the blockchain can guarantee completely that the BDS will not be hacked because of these cracking technology and computing power requirements.

## Framework design

**Figure 3:**
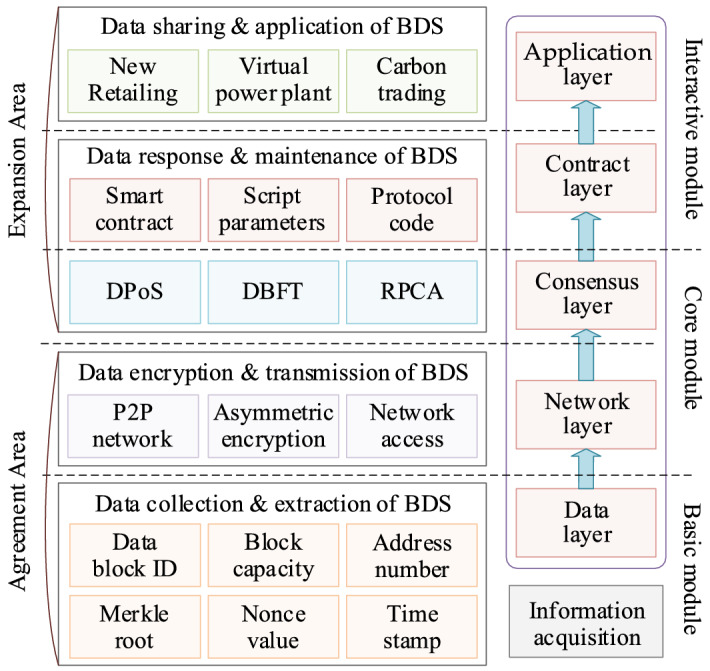
Framework structure of the enterprise BDS.

The enterprise BDS framework structure comprises a data layer, a network layer, a consensus layer, a contract layer, and an application layer (as illustrated in Fig. 3). Among these layers, the data layer, and the network layer are the basic modules and the required levels of the BDS. The consensus layer and the contract layer are the core modules and guarantee BDS operation, and the main system programming algorithms run on these layers. The application layer represents the interactive module between the BDS and the management personnel, and is the main level used to realize the enterprise digital functions.

### Data layer of the BDS

To resolve the semantic differences problem in the data characteristics, the BDS can use hybrid ontology-based information aggregation methods to clean and de-duplicate the enterprise data, slice the label data, and ultimately form a micro-ontology-based semantic structure^19^. With regard to the attribute differences issue in the data characteristics, the BDS can refer to the IEC61850 protocol to transform the text, pictures, URLs, and other information generated during enterprise operation into the more uniform formats of public information models, including the Common Information Models CIM/E, CIM/G, and CIM/XML. The block node in the data layer is composed of a block header and a block body. The block header mainly stores the block’s attribute information, including its identification number, timestamp, and Merkle root. The block body mainly stores specific data, including the data content, key instructions, the hash value, the address source, and the lock time, among other information.

**Figure 4:**
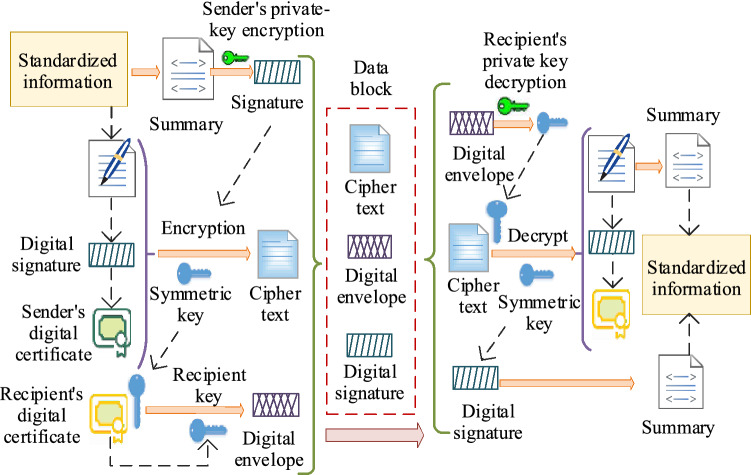
Asymmetric encryption process of the blockchain.

Before data are transmitted on the blockchain network layer, the standardized data must be encrypted within the block and connected in sequence in a time series to ensure data security^20^. Asymmetric data encryption is divided into three main steps (the specific steps are shown in Fig. 4), as per Step 1. The Merkle tree will then authenticate and classify the data in the block nodes rapidly to ensure that the hash algorithm can perform efficient operations on the data. In step 2, after the data are authenticated, the node block uses a hash function to encrypt the data asymmetrically and generate a public key, a private key, and a ciphertext. In step 3, the ciphertext, the key instructions, the address source, and the time stamp are sealed in the block node’s hyperledger and broadcast over the entire blockchain system. At this point, an asymmetric encryption cycle of the blockchain data system is complete.

### Network layer of the BDS

**Figure 5:**
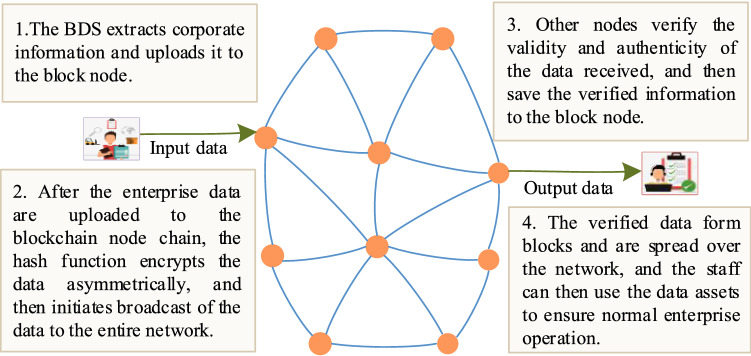
Data transmission process of the blockchain.

The BDS network layer contains technical elements that include the networking method for the block nodes and the data authentication protocol. The BDS network layer uses a P2P network for data transmission. In the network layer, each node not only undertakes network data transmission and block information authentication tasks, but also sets the routing protocol^21^. The data transmission process is illustrated in Fig. 5. The blockchain network layer typically has decentralization and distributed storage characteristics. The advantage of this P2P structure is that any node can authenticate, analyze, and store the enterprise data without relying on other nodes; as long as the number of failed or illegal nodes does not exceed 51% of the total number of nodes, then more than half of the computing power of the entire network will not be controlled, and the updating and extension of the main blockchain will remain unaffected.

The enterprise BDS uploads various collected data to the blockchain and broadcasts these data to the entire network’s nodes through the P2P network. When the other nodes receive new block information, they first verify the authenticity and validity of the data using the data structure, the key instructions, the address source, the time stamp, and other information. If the received information passes this verification stage, the block node will then store the data in the block body in accordance with the time sequence and continue to forward the data to its neighboring nodes. If the block receives illegal data, the chain network will immediately stop dissemination of these data to ensure that the illegal data will not be forwarded on the data platform.

### Consensus layer of the BDS

The consensus layer solves the information consensus and trust issues of the BDS, thus enabling some decentralized nodes to reach a consensus agreement rapidly for different data types, and also providing incentives for participants that make corresponding contributions^22^. In a highly autonomous distributed system, the combined application of multiple consensus mechanisms enables all block nodes to reach a consensus within a relatively short time. This is also the most critical factor in maintaining the fairness of the entire system and ensuring that the chain structure is not attacked using illegal data. Currently, blockchain includes seven main types of mainstream blockchain consensus algorithm (as shown in Table 2), and these different consensus mechanisms each have their own characteristics.

**Table 2:** Various consensus mechanisms used in the BDS.

S/N	Consensus mechanism	Abbre-viation	Features and application effects
1	Proof of work	PoW	The PoW enables all nodes in the digital system to maintain the corresponding competitive computing power required to participate in data processing and obtain block accounting rights. The PoW has strong security and reliability, but it takes a long time for a block to reach a consensus, with low work efficiency and high-power consumption
2	Proof of stake	PoS	The PoS optimizes the competitive computing powers of the block nodes, and distributes the accounting rights of the data blocks through the proportion of equity. The advantage of the PoS is that it reduces energy consumption, shortens the time required for block consensus, and improves the working efficiency of the BDS
3	Delegated proof of stake	DPoS	The DPoS only authorizes some of the digital system blocks, and these trusted authorized nodes take turns to maintain accounts and gradually form a new blockchain. The number of nodes that participate in authentication and accounting is greatly reduced, reducing the block node authentication time, saving power, and improving the system authentication efficiency
4	Proof of activity	PoA	The PoA is a hybrid algorithm that combines the algorithm advantages of workload proof and equity proof, reduces the operational complexity, shortens the calculation time from seconds to milliseconds, and can verify block information correctness in the shortest time
5	Practical Byzantine fault tolerance	PBFT	The PBFT requires all computing nodes participating in data authentication to maintain a specific state and perform the same actions. The PBFT can accommodate less than 1/3 of the total number of error block nodes. As long as more than 2/3 of the entire network participates in authentication, the BDS can operate normally
6	Delegated Byzantine fault tolerance	DBFT	The DBFT preferentially selects 2/3 of the dynamic participating nodes based on the block equity ratio, which can prevent most malicious attacks, enable the data block nodes to reach consensus rapidly, and shorten the authentication time. Additionally, to avoid a fork in the main chain of the block, this algorithm can accept any error type
7	Ripple proof of consensus algorithm	RPCA	The RPCA improves the data broadcasting algorithm to increase the speed of packaging, transmission, and authentication of the enterprise data in the block, allowing all nodes to confirm the data together, and improving the BDS information processing accuracy

### Contract layer of the BDS

The The BDS contract layer encapsulates the script parameters, the protocol codes, and the response rules for various smart contracts, and combination of smart contracts can enable the derivation of more complex contract commands^23^. Enterprise technicians embed the algorithms and the programming required to process data into the contract layer, ensuring that the entire BDS can analyze and mine the inherent value generated by the data automatically. Smart contracts have intelligent management and automatic operation characteristics that can trigger programming automatically to execute a contract according to the program code; the program is not subject to human control or to interference from external factors during operation. This feature improves the system’s data processing efficiency based on the premise of ensuring the independence of the contract layer.

**Figure 6:**
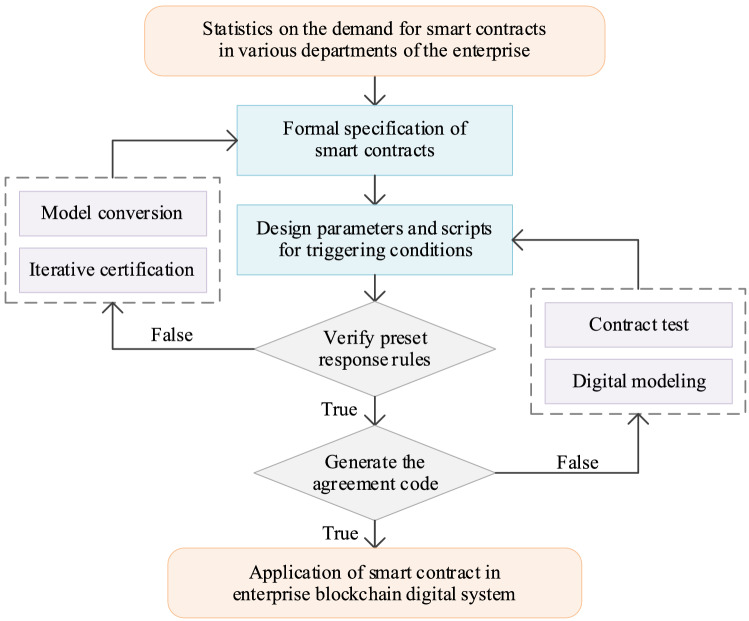
Data processing flow for smart contract.

In the initial BDS operation stage, the technical personnel of the enterprise draw up both the content of the smart contract and the activation conditions for contract execution, and then embed this content in the enterprise BDS in script code form. When the blockchain system is running, various data processing codes are embedded within specific blocks and are broadcast to all nodes via the blockchain network. When the smart contract meets the trigger conditions, it will then activate automatically and run the contract commands to complete data analysis, and this process is no longer affected by any other algorithms. The smart contract data processing flow is illustrated in Fig. 6.

### Application layer of the BDS

The BDS application layer encapsulates the BDS application scenarios and is also an interactive processing platform for the enterprise data. The BDS mainly analyzes and applies the enterprise’s static and dynamic data. Static data mainly include information with relatively low fluctuation frequencies, including company profiles, product information, and revenue data; dynamic data mainly include user details, contract lists, transaction information, and other information that fluctuates frequently, and the application values and application difficulty of these data will also vary proportionately. So, the BDS application layer can process distributed data in a timely manner, determine the current operating status of the enterprise rapidly, and produce a corresponding analysis report. Furthermore, all departments in the company can obtain relevant analysis reports through the block link port to complete their information interactions with the data system. In addition, the application layer can also use information feedback from the BDS to judge the requirements for enterprise improvement measures and assess the future development direction of the industry. The application layer not only provides a basic framework support for the digital system, but also provides technical services, operating models, and management strategies for the enterprise.

## Application scenarios

Fields that show strong demand for blockchain application usually involve the following distinctive features: fields in which the system’s internal communication efficiency is low, but the consensus is high; fields in which high data authenticity is required; and fields in which there is demand for high data flow efficiency and quality. Selection of the appropriate type of blockchain based on the characteristics of the different blockchain types can bring out the maximum efficiency of the BDS. Blockchain offers certain unique advantages, but it does not mean that a BDS can be applied in all industries. To achieve the perfect combination of enterprise and BDS, it is necessary to begin with the industry characteristics and match industries with similar functions to the blockchain. This study explores the characteristics and the industry applicability of blockchain from the perspectives of the three types of blockchain characteristics and selects three representative scenarios, e.g., E-retail supply chain, virtual power plants, and carbon trading platform, to analyze BDS operability fully in practical applications.

(1) Public blockchain is suitable for scenarios in which large numbers of subjects participate and do not require entry permission. Using E-retail supply chain as an example, the number of participants in the E-retail supply chain is huge, and the transaction behavior cannot be quantified regularly. Maintaining interactions with other nodes at any time will waste communication and computing resources. A BDS based on public blockchain can help participants to join the system when the transaction is in progress and then exit the system at a time when the transaction is stopped; this can record and protect the product production and transportation information effectively, and also reduces the computing costs and energy consumption of the BDS. (2) Consortium blockchain is suitable for scenarios in which a small number of participants must ensure operational efficiency. Using virtual power plant as an example, virtual power plants (VPP) are composed of various micro power generation systems and power transmission systems. A BDS based on consortium blockchain can help VPP to participate in power supply coordination management of the power market and grid operation. This can solve the problems of power shortage and low energy efficiency through off-peak power generation, and can achieve transparent and efficient energy aggregation optimization in an autonomous environment. (3) Private blockchain has become a technology that tends to be centralized and is suitable for data sharing within an organization to ensure that the data cannot be modified. Using the carbon trading platform as an example, a trading center can use a BDS based on private blockchain to audit and manage the carbon emission trading data of different enterprises across various regions. Based on the premise of ensuring that the data cannot be tampered with, this will improve the carbon trading information utilization rate and maximize the value of the data.

Based on the analysis above, industries that were relatively close to the characteristics of the three types of blockchain were selected in this study for in-depth analysis, with the aim of resolving the industry pain points with the aid of blockchain and the BDS platform.

### Application of BDS to E-retail supply chain

E-retail companies are using advanced technologies such as radio-frequency identification (RFID) and the IoT to transform the production, transportation, and sales processes of agri-food and other commodities, and are reshaping their business structures and ecological chains^24^. However, E-retail companies still face specific challenges, including product anti-counterfeiting traceability, logistics information forgery, consumer information leakage, and information transmission barriers. The probability of safety problems occurring within each link has thus increased greatly, and traceability and accountability difficulties are also increasing. Determination of how to ensure traceability information authenticity is thus a major problem for the entire E-retail industry. Specifically, when product quality disputes occur, it is difficult for traditional centralized databases to provide effective traceability evidence, but a BDS based on public blockchain can use its technical advantages to resolve these difficult problems for E-retail supply chains.

**Figure 7:**
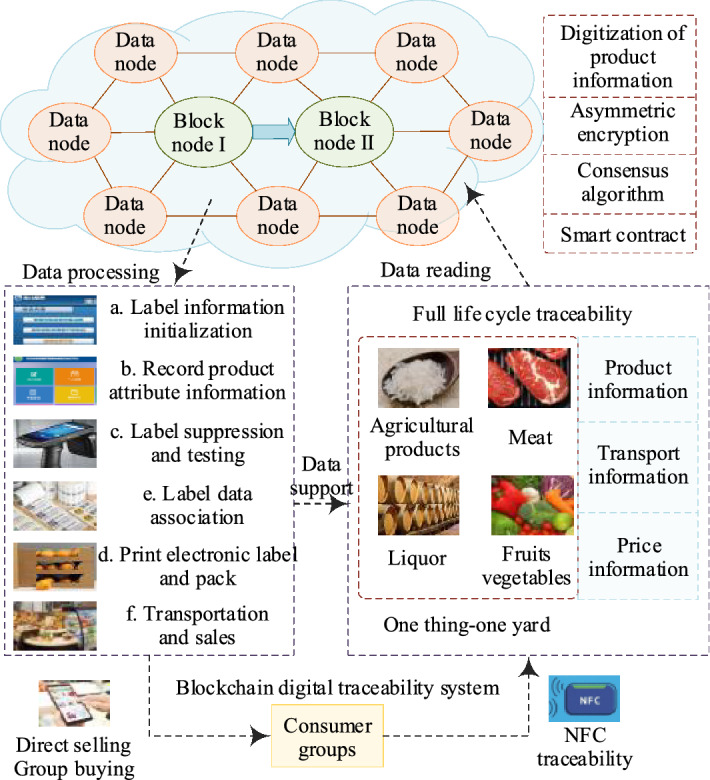
Operating procedure of the E-retail BDS.

The E-retail BDS process based on public blockchain operating is illustrated in Fig. 7. E-retail companies can implant RFID electronic tags at the beginning of product production, and the BDS will register the product information automatically. These products then have an electronic identity that allows the product’s full life-cycle information to be traced for anti-counterfeiting purposes. The information generated in each link throughout commodity processing, logistics, and warehousing is also recorded in the RFID tags through the BDS, which can then provide real information about each link to regulatory authorities, cooperative enterprises, and consumers^25^. Algorithm 1 demonstrates implementation of a smart contract for product registration; Algorithm 2 demonstrates the transactions updating at each step in the supply chain process.

- In Algorithm 1, the RFID reader reads the product tags and automatically obtains the product code and product name to register each product in the blockchain database. The smart contract first checks the manufacturer’s authorization from the blockchain ledger. If the authorization is passed, it checks whether the certified product code exists. If the code is found, then the manufacturer’s address, product code, product name, and timestamp are used to register the product in the blockchain ledger. If authentication fails, the counterfeit products data are not allowed to be saved in the blockchain ledger.
- In Algorithm 2, the transaction is updating, and when the recipient or consumer receives the product, a new transaction is added to the blockchain ledger containing the previous address of the product and its original manufacturer or supplier.

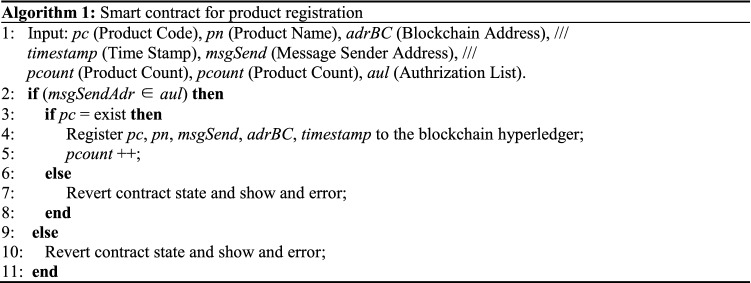



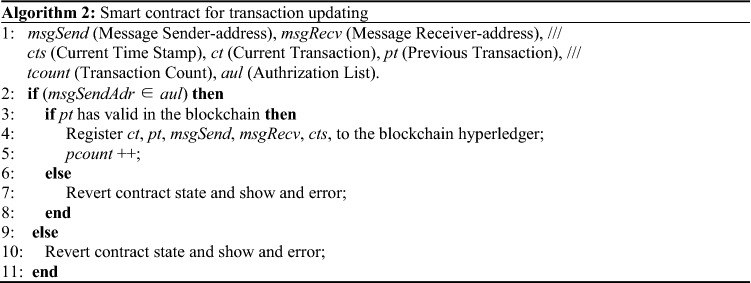


### Application of BDS to virtual power plants

**Figure 8:**
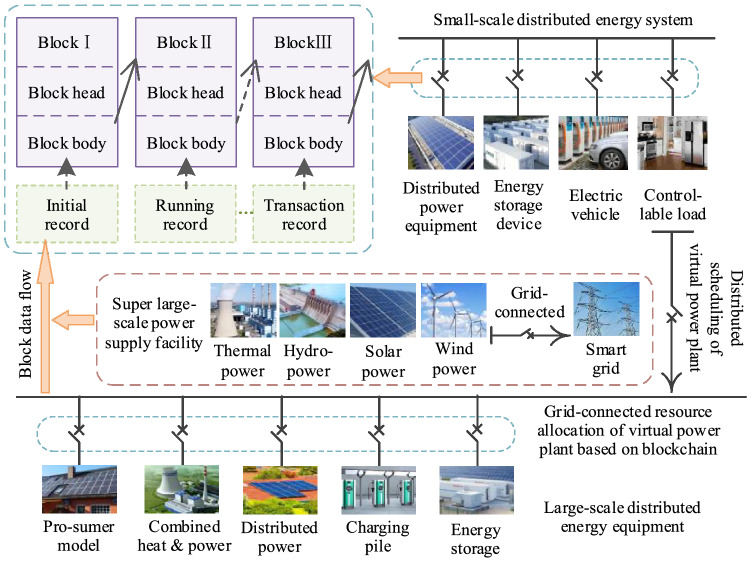
BDPS operation model of the VPP.

The virtual power plant (VPP) is currently the mainstream choice for use with distributed new energy power systems. The VPP is not restricted by geographical factors. Instead, the VPP uses a blockchain digital power system (BDPS) based on consortium blockchain to aggregate small and diverse distributed power sources, energy storage equipment, and the power IoT to form a special power plant and enable participation in operation of the power grid. Using the methods of peak-cutting and valley-filling, distributed energy can be transformed and consumed within the power system, thus reducing resource waste. The VPP, with the assistance of the BDPS based on consortium blockchain, uses P2P network communication technology to connect distributed power generation equipment (DPGE), combined heat and power (CHP) systems, the prosumer model (PSM), and power load platforms (PLPs) simultaneously, and can also record the real-time information and power status of the distributed power system^26^. Figure 8 illustrates the BDPS operation model based on the VPP.

In the VPP, the number of intelligent terminals configured for distributed energy system operations on different scales is also different. A VPP with fewer smart terminals can be connected to the grid directly through smart microgrids, while a VPP with more intelligent terminals can be coordinated and controlled with the aid of the BDPS based on consortium blockchain and participate in grid connection in a unified manner. This power block will be used as a basis for resource allocation and performance incentives to encourage more distributed energy companies to participate in grid connection activities, thus creating more grid connection opportunities and improving the grid connection efficiency and stability. Algorithm 3 demonstrates the smart contract that allows prosumers to complete initialization in the VPP^27^. Algorithm 4 shows the contract process for microgrid energy settlement.

- In Algorithm 3, the prosumers either consume the power in the VPP or provide power supply services to the VPP. Any prosumer can start a VPP initialization smart contract each time to ensure the fairness of opportunities for all prosumers in the process of establishing VPP.
- In Algorithm 4, the energy settlement process of VPP for prosumers is updated in real time. When the VPP construction session ends, the algorithm will return the first total energy quotation that can provide VPP expectations. If the total energy is greater than the energy required by VPP, the energy provided by the first few prosumers will be retained.

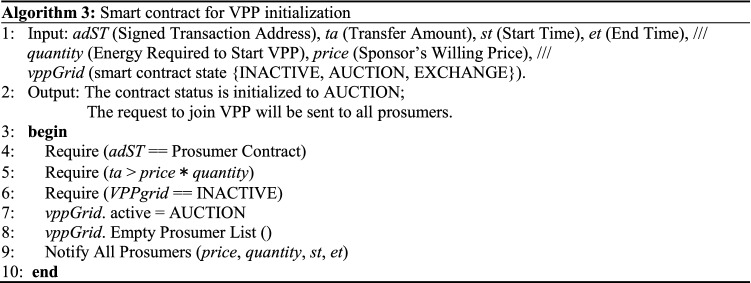



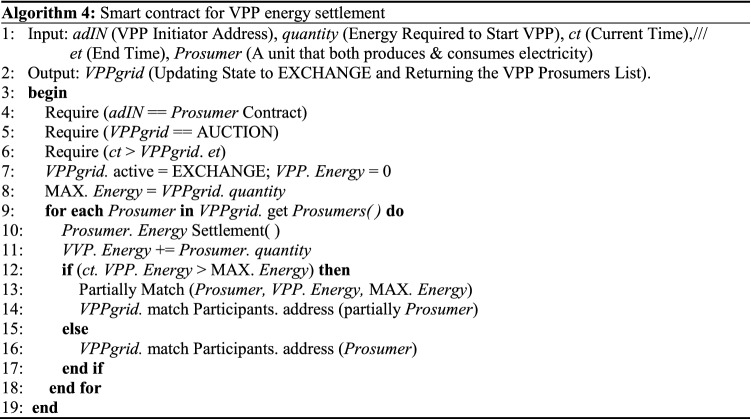


### Application of BDS to carbon trading platform

The principle of carbon trading is that the government incorporates enterprises with carbon emissions that have reached a designated scale into their carbon management system and then allocates annual carbon emission allowances to these enterprises annually. If the carbon emission allowance allocated to a company is not used up or is insufficient, the carbon trading platform then allows the company to use these carbon emission rights as a commodity in the market for trading^28^. A carbon trading platform based on private blockchain can convert carbon indicators into digital carrier values, allocate carbon emission rights in a reasonable manner, and encourage completion of carbon trading contracts. The information sharing and traceability characteristics of the BDS allow the carbon trading platform to track and record allocations of carbon allowances, thus providing a credible and reliable regulatory environment for carbon trading.

**Figure 9:**
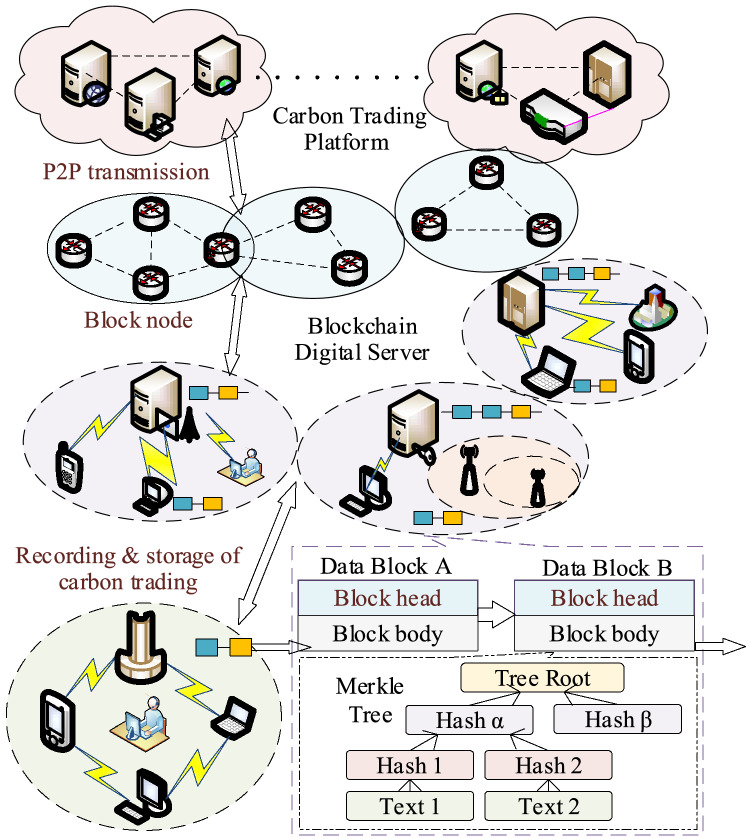
Framework of carbon trading platform based on BDS.

The framework for a carbon trading platform based on a BDS is shown in Fig. 9. With the support of the BDS, the carbon trading platform cannot perform excessive external interventions in the trading business, but is specifically responsible for trading security and platform maintenance. Smart contracts can record all transaction contracts and settlement lists, which are stored permanently on the server and cannot be modified at will. This not only simplifies the carbon trading platform system settings and reduces its operating costs, but also resolves the problems of low-efficiency multi-agent identity authentication and data confirmation difficulties during the trading process. Additionally, private blockchain allows the transaction parties to maintain distributed data jointly, thus increasing carbon trading transparency, stabilizing carbon market prices, and improving business processing efficiency^29^. Algorithm 5 illustrates the transaction data uploading process of the carbon trading platform. Algorithm 6 illustrates the process of querying historical logs in the carbon trading platform.

- In Algorithm 5, if the information parameters meet carbon trading platform index requirements, the smart contract will package and upload in accordance with the current carbon trading data, and return the message “Success”. If these requirements are not met, a “Carbon trading data exception warning” prompt will appear. Consequently, an exception value will be returned. If the production indicators do not meet the quality requirements, a “Carbon trading contract ID record exception” prompt will appear, and the contract will be terminated.
- In Algorithm 6, different nodes call the data query contract by inputting the carbon trading information. After user permission is confirmed and the authentication is successful, the contract will call the corresponding module to query the information in accordance with the retrieval type and record the querier’s information and time. In contrast, if the corresponding information is not found, a “No carbon trading query record” message will be returned. If the authorization verification is unsuccessful, the “User authorization verification failed” message will be returned.

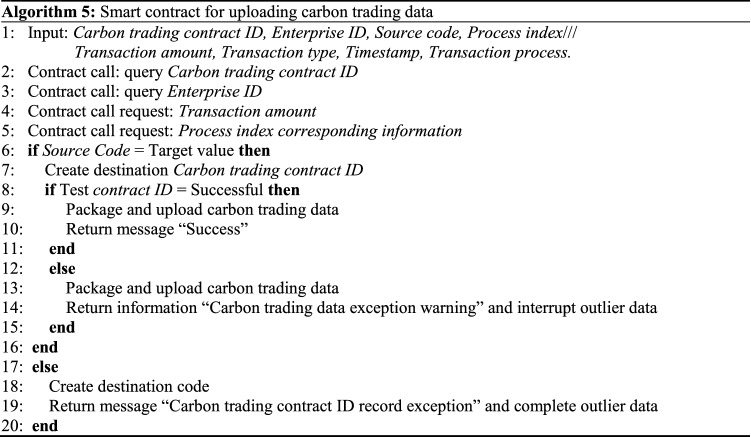



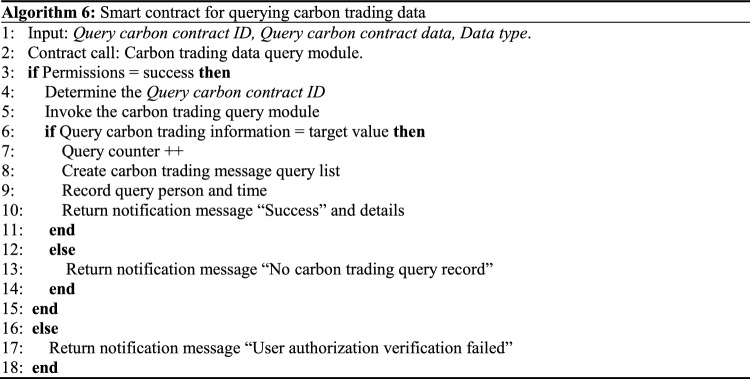


## Conclusion

This article introduces blockchain into digital management processes with the aim of breaking down barriers in enterprise digitization processes by using advantageous technologies such as distributed storage and encrypted transmission. This article proposes a model BDS architecture and analyzes the operating mechanism on each level in detail. At the end of the study, the author discusses the potential advantages of massive data and rich application scenarios, and demonstrates how three different industry types can achieve digitization using blockchain. The introduction of blockchain into the digital transformation of enterprises is still in its initial exploration period, and a relatively uniform system standard has not been established to date in the academic field. Blockchain should not be limited to Bitcoin mining and currency speculation, but should also be used with existing technologies to solve practical problems. With continuous promotion of blockchain, its potential application scenarios will become increasingly abundant. As the basic framework for industry digitization, the BDS will inevitably attract increasing attention. This article hopes to offer beneficial results for future applications of blockchain technology (Supplementary Information).

## Supplementary Information

Supplementary Information.

## Data Availability

Data sets (source code, raw data) generated and analyzed during the current study are available in the github.com repository, https://github.com/sw8258/BDSsourcecode.git and also are available from the corresponding author on reasonable request.
