# Supplementary material for: Research on model design and operation mechanism of enterprise blockchain digital system
Source: Sci Rep. 2022 Nov 24;12:20286. doi: 10.1038/s41598-022-24796-0 (PMC9700752; doi:10.1038/s41598-022-24796-0)
Supplement: Supplementary file 1 — Supplementary Information. [file 41598_2022_24796_MOESM1_ESM.pdf]

Supplementary Information

Table 1. Characteristics of the three blockchain types.

S/N	Public blockchain	Consortium blockchain	Private blockchain
**Openness**	Fully public	Partly public, partly encrypted	Fully encrypted
**Degree of centralization**	Fully decentralized	Retains some centralized functions	Fully centralized
**Scope of application**	Widest	Wider	Narrowest
**Participants**	Anyone	Pre-designated personnel only	Managers only
**Data read and write**	All participants	Persons with special permissions only	Managers only
**Confidentiality**	Weakest	Strong	Strongest
**Features**	Completely decentralized, reliable, and data are open, transparent, and unforgeable.	Partially decentralized, ability to set up special management authority, strong scalability, high information processing speed, and low transaction costs.	Easy to control, convenient rule modification, and offers efficient management.
**Application scenarios**	Virtual currency transactions	Digital transformation of enterprises, and information transmission, storage, and management	Accounting audit

Table 2. Various consensus mechanisms used in the BDS.

	Consensus mechanism	Abbre-viation	Features and application effects
1	Proof of work	PoW	The PoW enables all nodes in the digital system to maintain the corresponding competitive computing power required to participate in data processing and obtain block accounting rights. The PoW has strong security and reliability, but it takes a long time for a block to reach a consensus, with low work efficiency and high-power consumption.
2	Proof of stake	PoS	The PoS optimizes the competitive computing powers of the block nodes, and distributes the accounting rights of the data blocks through the proportion of equity. The advantage of the PoS is that it reduces energy consumption, shortens the time required for block consensus, and improves the working efficiency of the BDS.
3	Delegated proof of stake	DPoS	The DPoS only authorizes some of the digital system blocks, and these trusted authorized nodes take turns to maintain accounts and gradually form a new blockchain. The number of nodes that participate in authentication and accounting is greatly reduced, reducing the block node authentication time, saving power, and improving the system authentication efficiency.
4	Proof of activity	PoA	The PoA is a hybrid algorithm that combines the algorithm advantages of workload proof and equity proof, reduces the operational complexity, shortens the calculation time from seconds to milliseconds, and can verify block information correctness in the shortest time.
5	Practical Byzantine fault tolerance	PBFT	The PBFT requires all computing nodes participating in data authentication to maintain a specific state and perform the same actions. The PBFT can accommodate less than 1/3 of the total number of error block nodes. As long as more than 2/3 of the entire network participates in authentication, the BDS can operate normally.
6	Delegated Byzantine fault tolerance	DBFT	The DBFT preferentially selects 2/3 of the dynamic participating nodes based on the block equity ratio, which can prevent most malicious attacks, enable the data block nodes to reach consensus rapidly, and shorten the authentication time. Additionally, to avoid a fork in the main chain of the block, this algorithm can accept any error type.
7	Ripple proof of consensus algorithm	RPCA	The RPCA improves the data broadcasting algorithm to increase the speed of packaging, transmission, and authentication of the enterprise data in the block, allowing all nodes to confirm the data together, and improving the BDS information processing accuracy.

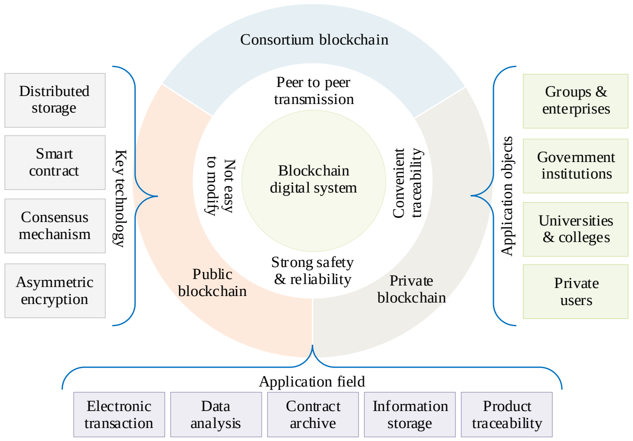

Figure 1. Attribute characteristics of blockchain.

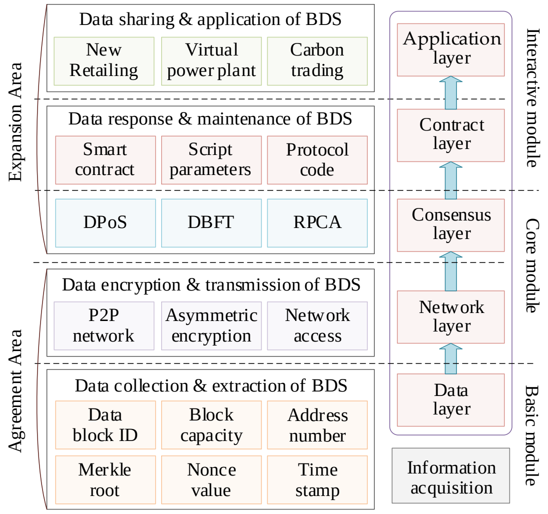

Figure 3. Framework structure of the enterprise BDS.

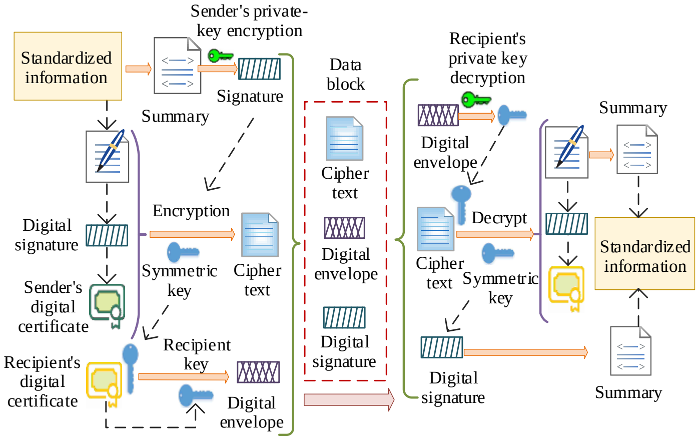

Figure 4. Asymmetric encryption process of the blockchain.

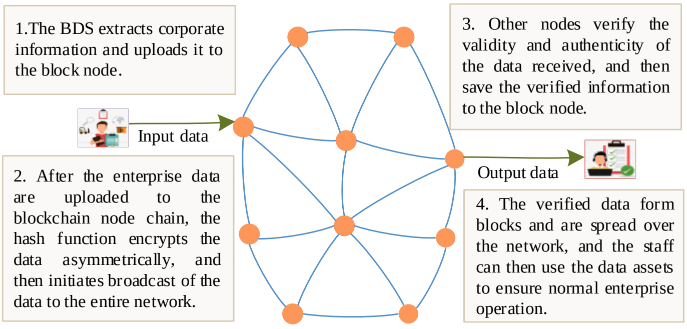

Figure 5. Data transmission process of the blockchain.

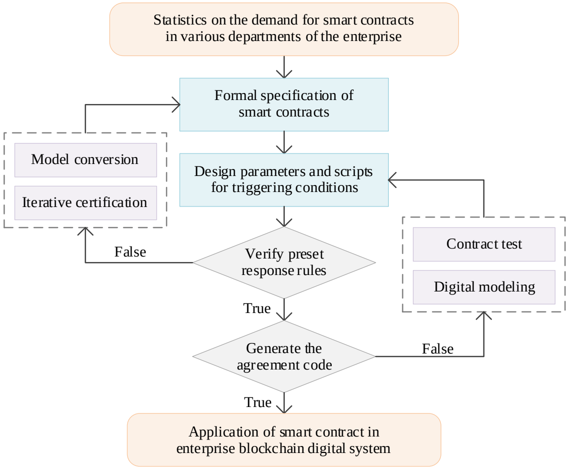

Figure 6. Data processing flow for smart contract.

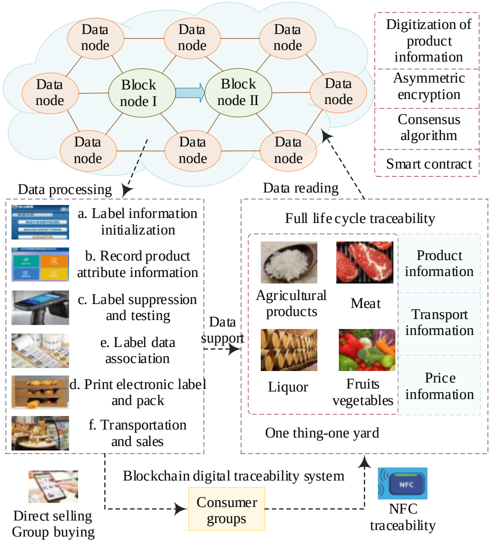

Figure 7. Operating procedure of the E-retail BDS.

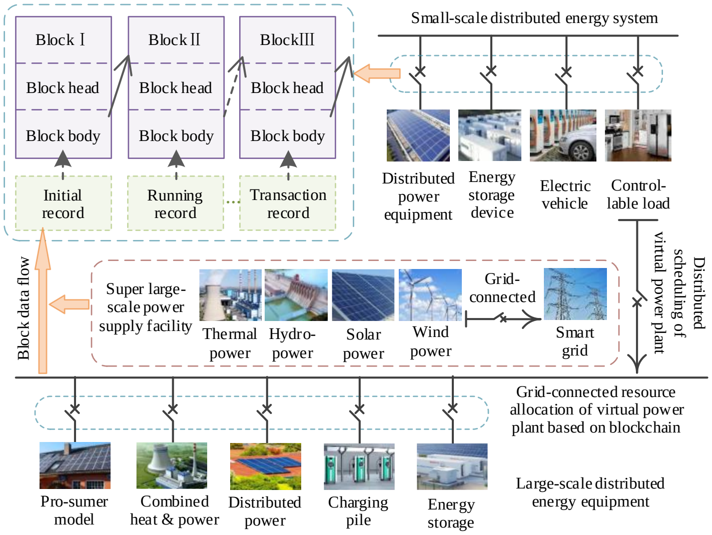

Figure 8. BDPS operation model of the VPP.

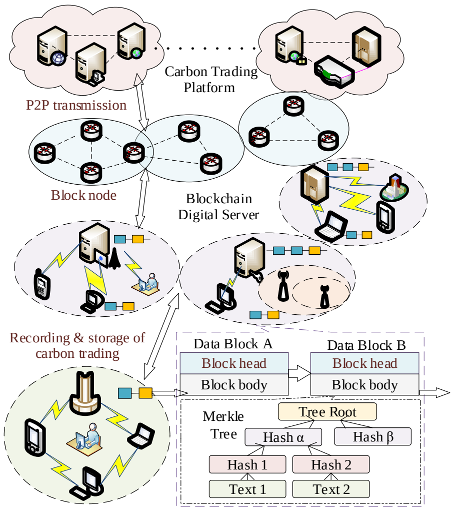

Figure 9. Framework of carbon trading platform based on BDS.

The original code of “Data tamping success rate” and the generation process of Fig.2.

1. Calculation formula of BDS data stamping success rate:

$$\mathrm{P}_n = \begin{cases} (\frac{q}{p})^n, & p > q \\ 1, & p \le q \end{cases} \tag{1}$$

$$\lambda = n\frac{q}{p} \tag{2}$$

$$\mathrm{P}_\xi = \sum_{k=0}^{\infty} \frac{\lambda^k e^{-\lambda}}{k\,!} \cdot \begin{cases} (\frac{q}{p})^{n-k}, & k \le n \\ 1, & k > n \end{cases} \tag{3}$$

2. Input the following code in the “Maple” software:

```maple```

```
Ps:=proc(z, q, cnt)
    local(lam, p, fun):
    fun:=proc(z, q, k)
        if k <= z then
            return (q/(1 - q))^(z - k)
        else
            return 1;
        fi;
    end proc;
    lam := z*q/(1 - q);
    return add(lam^k*exp(-lam)*fun(z, q, k)/k!, k = 0 .. cnt);
end proc:
z:= 8 :
 q:= 0.3:
 cnt:= 1000:
Ps(z, q, cnt);
```
```

3. Output the result of the BDS data tampering success rate:

Gap between blocks	q=0.1	q=0.2	q=0.3	q=0.4	q=0.5
0	1	1	1	1	1
1	0.20459	0.4159	0.62775	0.82886	1
2	0.05098	0.20393	0.44572	0.7364	1
3	0.01317	0.10324	0.32458	0.66417	1
4	0.00346	0.053	0.23913	0.6034	1
5	9.14E-04	0.02742	0.17735	0.55063	1
6	2.43E-04	0.01425	0.13211	0.50398	1
7	6.47E-05	0.00743	0.09871	0.4623	1
8	1.73E-05	0.00389	0.07392	0.42478	1
9	4.63E-06	0.00203	0.05546	0.39083	1
10	1.24E-06	0.00107	0.04166	0.35998	1
11	0	5.60E-04	0.03133	0.33186	1
12	0	2.94E-04	0.02358	0.30617	1
13	0	1.55E-04	0.01777	0.28265	1
14	0	8.14E-05	0.01339	0.26108	1
15	0	4.29E-05	0.0101	0.24128	1
16	0	2.26E-05	0.00762	0.22308	1
17	0	1.19E-05	0.00575	0.20632	1
18	0	6.27E-06	0.00435	0.1909	1
19	0	3.31E-06	0.00328	0.17668	1
20	0	1.74E-06	0.00248	0.16356	1

4. Output **Fig.2** through “Originlab” :

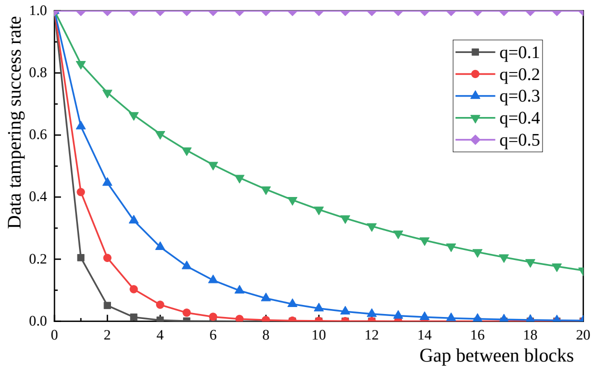

Figure 2. Data tampering success rate characteristics for the BDS.
